# Technical Analysis of cDNA Microarrays

**DOI:** 10.1371/journal.pone.0004486

**Published:** 2009-02-16

**Authors:** Cinda P. Scott, Jeff VanWye, M. Danielle McDonald, Douglas L. Crawford

**Affiliations:** Department of Marine Biology and Fisheries, Rosenstiel School of Marine and Atmospheric Science, University of Miami, Miami, Florida, United States of America; University of Edinburgh, United Kingdom

## Abstract

**Background:**

There is extensive variation in gene expression among individuals within and between populations. Accurate measures of the variation in mRNA expression using microarrays can be confounded by technical variation, which includes variation in RNA isolation procedures, day of hybridization and methods used to amplify and dye label RNA for hybridization.

**Methodology/Principal Findings:**

In this manuscript we analyze the relationship between the amount of mRNA and the fluorescent signal from the microarray hybridizations demonstrating that for a wide-range of mRNA concentrations the fluorescent signal is a linear function of the amount of mRNA. Additionally, the separate isolation, labeling or hybridization of RNA does not add significant amounts of variation in microarray measures of gene expression. However, single or double rounds of amplification for labeling do have small but significant affects on 10% of genes, but this source of technical variation is easy to avoid. To examine both technical and stochastic biological variation, mRNA expression was measured from the same five individuals over a six-week time course.

**Conclusion:**

There were few, if any, meaningful differences in gene expression among time points. Thus, microarray measures using standard laboratory procedures can be precise and quantitative and are not subject to significant random biological noise.

## Introduction

Microarrays simultaneously quantify several hundred to thousands of genes on a single glass slide and their use has greatly expanded the breadth of quantified gene expression [1]–[10]. Yet the preparation of RNA affects the precision of microarray measures and therefore the ability to accurately quantify the content of an RNA sample [11]. Additionally, differences in microarray platforms, laboratory procedures and post-quantification analyses affect the precision among arrays [12]–[15]. Thus, technical variation can substantially affect the interpretation of microarrays.

For the teleost fish *Fundulus heteroclitus* variation among individuals in mRNA expression is extensive: >60% of genes have significant differences in expression among individuals within a population [1], [9], [16], [17]. Many of these differences in gene expression are associated with variation in cardiac metabolism [9]. However, the accuracy and biological relevance of these differences in expression depends on the technical variation inherent to microarray processing [1].

Accurate microarray quantification is best realized when there is a linear relationship between fluorescence and RNA concentration. This linear relationship fails when the dynamic range of microarrays are exceeded. For any microarray, there are two parameters that define its dynamic range: the range of fluorescence that can be measured and the range of RNA concentrations that can bind to a specific array feature. These two components of the dynamic range reflect the two types of saturation that can occur on a microarray: photomultiplier tube (PMT) saturation and biological saturation. A linear relationship between fluorescence and RNA concentration can only occur if the cDNA on the microarray captures proportional amounts of RNA and if the PMT is not saturated.

The PMT measures the number of photons from the fluorescently labeled RNA that are excited by the lasers. PMT saturation is a result of the photomultiplier tube becoming oversaturated due to an overabundance of converted electrons by the analog to digital (A/D) converter. The A/D converter can only convert the PMT signal into a value less than or equal to 2^16^-1 or 65,535 and thus any fluorescent photons captured at this value of 65,535 are not discernable [18]. This type of saturation can be avoided by reducing the PMT voltage and laser power. Alternatively, the specific activity of the mRNA (number of fluorescent molecules per message) can be reduced. However, the reduction of the PMT voltage, power of the lasers, or reduced labeling, does not address the question of whether or not a particular cDNA on a microarray is biologically saturated.

Biological saturation occurs when the amount of mRNA that can hybridize to the DNA on a microarray reaches a maximum binding capacity of the printed DNA. If biological saturation is reached, then the amount of a mRNA will be underestimated and differences among arrays or experiments can not be appropriately determined. To avoid biological saturation, the amount of target RNA must be present in quantities less than the amount that the cDNA on the microarray slide can bind. To determine the range and linear response of increasing amounts of mRNA, we hybridized a 500-fold concentration range of labeled RNA from cardiac tissue to the *F. heteroclitus* 384 cDNA metabolic microarray.

Sources of technical variation, other than PMT and biological saturation, come from methods used to fluorescently label the mRNA, the day on which the RNA is processed and varying amounts of available tissue [19], [20]. One of the most common approaches to fluorescently label mRNA for microarray studies is to amplify the RNA by synthesizing cDNA with a T7 RNA polymerase binding site. RNA is then synthesized *in vivo* by using the T7 RNA polymerase to incorporate amino allyls followed by covalent binding of fluorescent molecules to the incorporated amino allyls [21]. For small amounts of starting mRNA, the synthesis of RNA using T7 can be repeated to double the amplification. To understand the effect of a single round versus a double round of linear amplification we compared the quantification of RNA using both methods.

The day and process used to isolate mRNA are two additional sources of technical variation. Variation in the preparation of mRNA could alter its quality affecting how well the RNA amplifies, is fluorescently labeled, and the signal observed on the microarray. The day on which a tissue is sampled is not strictly technical but can introduce a second type of variation: biological variation. That is, isolating tissues on different days could introduce technical variation because of the precision of dissection and the quality of tissue or RNA preparation. However, because tissues are sampled on different days, the organisms may be biologically different (under more or less stress, healthier, or just one day older). To examine technical variation due only to RNA isolation, a single blood sample was divided into four, RNA was separately isolated from each sample and, gene expression was quantified. Biological variation was examined in a separate experiment where five fish were bled every two weeks for a total of six weeks in order to collect four separate samples from each individual. Gene expression was quantified for these four temporally separate samples.

These experiments indicate that for a wide range of experimental conditions, microarray experiments using the *Fundulus* array are both accurate and precise.

## Materials and Methods

### Organism

*Fundulus heteroclitus* were caught from wild populations in Wiscasset, Maine, USA (43°57′41″N, 69°42′45″W) by minnow trap. Fish were transported to the Rosenstiel School of Marine and Atmospheric Science at the University of Miami and acclimated to 20°C and 15ppt for approximately 6 months.

#### Blood Sampling

*Fundulus heteroclitus* (*N* = 20) were anesthetized with MS222 (0.1 g·l^−1^) and given tags with subdermal latex markers. Whole blood samples from each fish were taken every two weeks by caudal puncture using a 50 µl Hamilton syringe rinsed with heparinzed saline (50 i.u. ·ml^−1^). Samples were immediately frozen in liquid N_2_ and stored at −80°C. Only individuals that had all four serial samples taken (*N* = 5) were used in the present study.

### RNA isolation and amino allyl labeling

Total RNA was isolated using 4.5 M guanidinium thiocyanate, 2% N-lauroylsarcosine, 50 mM EDTA, 25 mM Tris-HCl, 0.1 M β-Mercaptoethanol and 2% Antifoam A. The extracted RNA was further purified using a Qiagen RNeasy Mini kit in accordance with the manufacturer's protocols. The quantity and quality of the RNA was determined using a spectrophotometer (Nanodrop, ND-1000 V3.2.1) and a bioanalyzer (Agilent 2100). RNA was then converted into amino allyl labeled RNA (aRNA) using the Ambion Amino Allyl MessageAmp II aRNA Amplification kit. This method converts poly-A RNA into cDNA with a T7 RNA polymerase binding site; T7 is then used to synthesize new strands of RNA (*in vitro* transcription)[22]. During this *in vitro* transcription of aRNA, an amino allyl UTP (aaUTP) is incorporated into the elongating strand. aaUTP incorporation allows for the coupling of Cy3 or Cy5 dyes (GE biosciences) onto aRNA for microarray hybridization.

Dye labeled aRNA aliquots for each hybridization (typically 30 pmol each of Cy3 and Cy5) were vacuum dried together and resuspended in 15 µl hybridization buffer (final concentration of each labeled sample = 2 pmol/µl). Hybridization buffer consisted of 5× SSPE, 1% SDS, 50% formamide, 1 mg/ml polyA, 1 mg/ml sheared herring sperm carrier DNA, and 1 mg/ml BSA. Slides were washed in sodium borohydride solution in order to reduce autofluorescence. Following rinsing, slides were boiled for 2 minutes and spin-dried in a centrifuge at 800 rpm for 3 minutes. Samples (15 µl) were heated to 90°C for 2 minutes, quick cooled to 42°C, applied to the slide (hybridization zone area was 350 mm^2^), and covered with a cover slip. Slides were placed in an airtight chamber humidified with paper soaked in 5× SSPE and incubated 24–48 hours at 42°C.

### Microarrays

mRNA expression was measured using microarrays where each array had four spatially separated replicates per gene. The 384 *F. heteroclitus* cDNA microarrays were printed using 55 control genes and 329 cDNAs which encode essential proteins for cellular metabolism (Table 1). The annotation of genes and related pathways used *FunnyBase* [23] and these were manually compared to KEGG pathway designations. Because many genes belong to more than one pathway, central metabolic pathways were preferentially used if the gene coded for a protein that was a catabolic or anabolic enzyme (*versus* acting in a signaling pathway that affected metabolism). Controls include DNA spots labeled with Cy5 (positive control for position and gridding) and *Ctenophore* cDNA as negative controls.

**Table 1:** 384 Array Metabolic Pathways.

Amino acid metabolism	28
ATP synthesis	27
Blood group glycolipid biosynthesis	3
Channel	3
Citrate cycle (TCA cycle)	24
Fatty acid metabolism/transport	36
Fructose and mannose metabolism	4
Galactose metabolism	2
Glutamate metabolism	7
Glutathione metabolism	10
Glycerolipid metabolism	7
Glycolysis/Gluconeogenesis	27
Inositol phosphate metabolism	14
Ox-Phos-ATPsyn	64
Pentose phosphate pathway	6
Purine & Pyrimidine metabolism	9
Pyruvate metabolism	2
Signaling pathway	10
Starch and sucrose metabolism	2
Sterol biosynthesis	8
Synthesis and degrad. of ketone bodies	4
Tetrachloroethene degradation	3
Secondary	27
TOTAL METABOLIC GENES	329

Microarrays were created by printing cDNAs amplified with amine-linked primers onto 3-D Link Activated slides (Surmodics Inc., Eden Prairie, MN) at the University of Miami's microarray facility. All printed cDNAs were re-sequenced from the same source used for printing. The microarray slides were scanned using ScanArray Express. The raw TIFF-image data was quantified using Imagene (v5).

All experiments used a loop design for hybridization of dye labeled aRNA [24], [25]. In a loop design [24], [25] each individual is labeled with Cy3 and Cy5. Each dye labeled sample is then hybridized on different arrays with another individual [26]. Thus, each individual is hybridized to two arrays with four replicates per array for a total of eight technical replicates per individual. This experimental design is a more efficient use of resources, providing more data per array and is thus statistically more powerful than a reference design.

To test for the relationship between fluorescence and the quantity of RNA, five concentrations of fluorescently labeled RNA were used: 1.2 to 700 pmol of Cy3 or Cy5 labeled mRNA where pmol are for the amount of incorporated dye (Table 2). A 15 µl hybridization using the 384 cDNA array corresponds to 0.09 to 47 µM of Cy dye. Cy5 dye labeled RNA was used at concentrations 18% less than Cy3 because the Cy5 dye is a more efficient fluorophore (greater fluorescence per photon) than the Cy3 dye. The average of eight fluorescence values for each gene was normalized to the original concentration of RNA added.

**Table 2:** Concentrations of Cy3 and Cy5 dye labeled RNA used for hybridization.

	50×	10×	5×	1×	.1×
Cy 3	700 pmol	140 pmol	70 pmol	14 pmol	1.4 pmol
Cy 5	583.3 pmol	116.6 pmol	58.3 pmol	11.6 pmol	1.2 pmol

### Criteria for Inclusion

For a gene to be included in an analysis, the average signal among all arrays and dyes had to exceed background but not exceed 95% of PMT saturation (65,535). Background signal was determined as the amount of fluorescence in negative control array elements. Not all genes met these criteria and therefore were not included in the analysis.

### Statistics

To adjust for systematic variation, gene expression values were first sum normalized, log2 transformed, and then loess normalized using Microarray Data Analysis System Software (MIDAS) [12], [27] and SAS JMP Genomics v.6.0.2. For every gene, eight fluorescence values were captured; four Cy3 values and four Cy5 values. Analysis of variance (ANOVA) was performed using SAS JMP Genomics v.6.0.2. To look for differences between single and double rounds of amplification the following ANOVA model was applied: y*_ijkl_* = μ+A*_i_*+D*_j_*+*_k_*+R*_l_*+ε*_ijkl_* where μ is the sample mean, A*_i_* is the effect of the *i* ^th^ array (*i* = 1–18), D*_j_* is the effect of the *j* ^th^ dye (Cy3 or Cy5), T*_k_* is the effect of the number of rounds of amplification (single or double, *k* = 2), R*_l_* is the effect of the day on which samples were prepared (*l* = 3), and epsilon is stochastic effects. The number of rounds of amplification (single or double) and channel variables were treated as fixed effects and array, and day on which samples were prepared were treated as random effects. Statistical analyses of replicate blood samples or repetitive measures of the same five individuals were applied to a separate ANOVA for each individual. The ANOVA model for this comparison was as follows: y*_mnp_* = μ+A*_m_*+D*_n_*+T*_p_*+ε*_mnp_* where μ is the sample mean, A*_m_* is the effect of the *m* ^th^ array (m = 1–4 for both replicate and repetitive samples), D_n_ is the effect of the *n* ^th^ dye (Cy3 or Cy5), T*_p_* is the treatment effect and epsilon is stochastic effects. Sample, representative of either one of four temporal samples from an individual or one of four replicate blood samples, and channel were treated as fixed effects. Array was treated as a random effect. Significant differences were evaluated with a p-value cut-off of 0.01.

## Results

### Biosaturation

The concentration of fluorescently labeled RNA (0.09 to 47 µM of Cy dye) represents 0.1×, 1×, 5×, 10×, 50× the concentration of RNA typically used on *F. heteroclitus* cDNA microarrays [9], [26], [28]–[30] (Table 2, MIAME GSE12858). Among the 329 metabolic genes on the array, 212 of these genes met our criteria of being less than 95% of the PMT saturation and more than two standard deviations above the negative controls (*Ctenophore* cDNA with no similarity to vertebrate genes).

**Figure 1:**
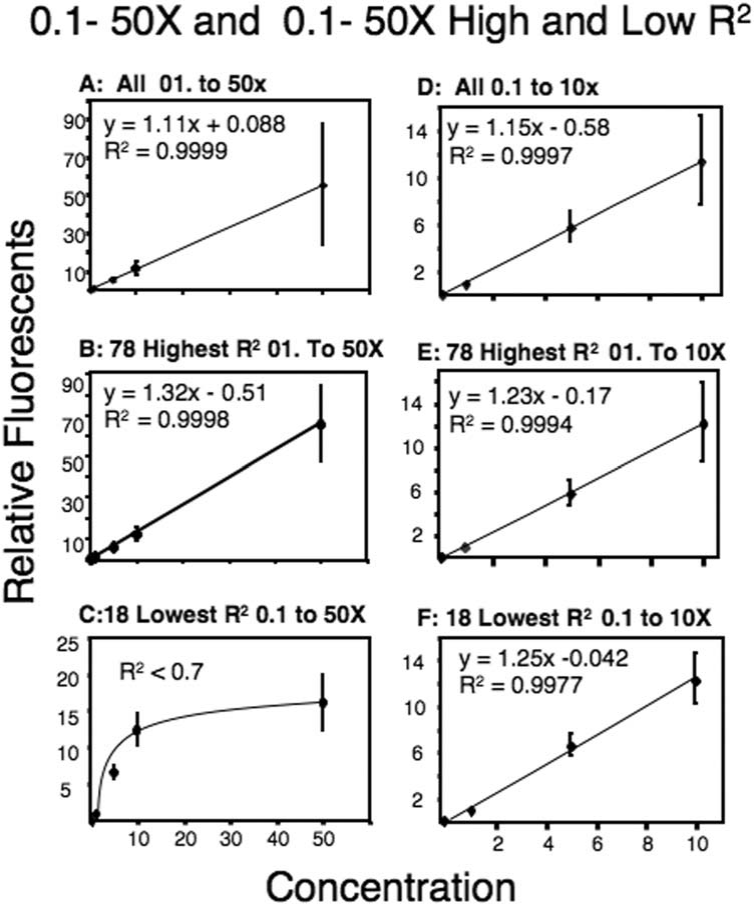
Linear relationship of RNA concentration to relative fluorescence. Graphs show linear relationship between concentrations of RNA (0.1–50×, A–C, and 0.1–10×, D–F) and relative fluorescence. Relative fluorescence is a normalized measure of fluorescence divided by the gene specific mean. 1× RNA is equal to 0.9 pmol/µl. Shown are the RNA concentrations *versus* fluorescence for 0.1 to 50× (A–C) and for 0.1× to 10× (D–F); for all genes (A and D), for the 78 genes with the highest R^2^ values (B and E), and for the 18 with lowest R^2^ values (C and F).

The linear relationship between the amount of RNA and relative fluorescence is shown in Figure 1. To remove the gene specific differences in expression, the fluorescence at each concentration was divided by the mean fluorescence for that specific gene (Fig. 1). The linear relationship between the amount of total fluorescent RNA added and the measures of gene specific fluorescence was determined for each gene. Most genes (176/212 or 83%) had an R^2^>95% and 78 genes had a nearly perfect R^2^ (>0.995; Fig. 1B; Table 3). Examining the 18 genes with the lowest R^2^ values (less than 0.8) revealed a non-linear relationship that can be explained by an apparent saturation at the 50× concentration of RNA (Fig. 1C). The relationship disappears if the fluorescence values for the 50× concentrations of RNA are removed and the 0.1 to 10× are plotted (Fig 1D–F). In the 100-fold range (0.1 to 10×) only three genes (1.4%) had R^2^ values less than 0.8 (Table 3). Examination of the higher concentrations (1.0 to 50×) revealed 19 genes (9%) with R^2^ less than 0.8 (Table 3). These data suggest that for most genes there is a linear relationship for a 500-fold range of RNA, however some cDNAs on the microarray will reach biological saturation at the highest RNA concentration.

**Table 3:** Number of genes and corresponding R^2^ for various ranges of RNA concentrations.

R^2^	0.1–50×	0.1–10×	1.0–50×
>0.9	176	199	178
<0.8	18	3	19

### Variation in RNA preparation

To determine how RNA preparation affects variation, cardiac RNA from three individuals were combined, and then evenly divided and amino allyl and dye labeled on three separate days using single and double rounds of amplification (MIAME, GSE12858). Only 110 genes met our criteria for inclusion because many genes were below the low cut-off (*Ctenophore* negative control cDNAs). In this experiment fewer genes met our criteria of above background and below saturation due to sample RNA being divided for separate labeling using either single and double rounds of amplification. An ANOVA was performed to measure differences between single and double rounds of amino allyl labeled RNA amplification. Twelve of the 110 genes (11%) used in this analysis were significantly different between single and double rounds of amplification at p<0.01. The majority of genes (59%) had a higher fluorescence signal when only one round of amplification was performed.

### Consistency of Quantitative Determination

In teleost fish, red blood cell (RBCs) nuclei are transcriptionally active [31], [32], and these cells can be sampled without sacrificing the fish. Thus to assess the consistency of microarray determinations, two experiments were performed on blood gene expression: 1) to examine technical variation a single sample of blood was divided into four samples; RNA isolations, amino allyl and dye labeling, hybridization and quantitative analyses were performed on each sample and 2) to examine biological variation, RNA isolated from blood from the five individuals were each sampled four times over a 6 week period (two weeks between samples; MIAME, TBA).

**Figure 2:**
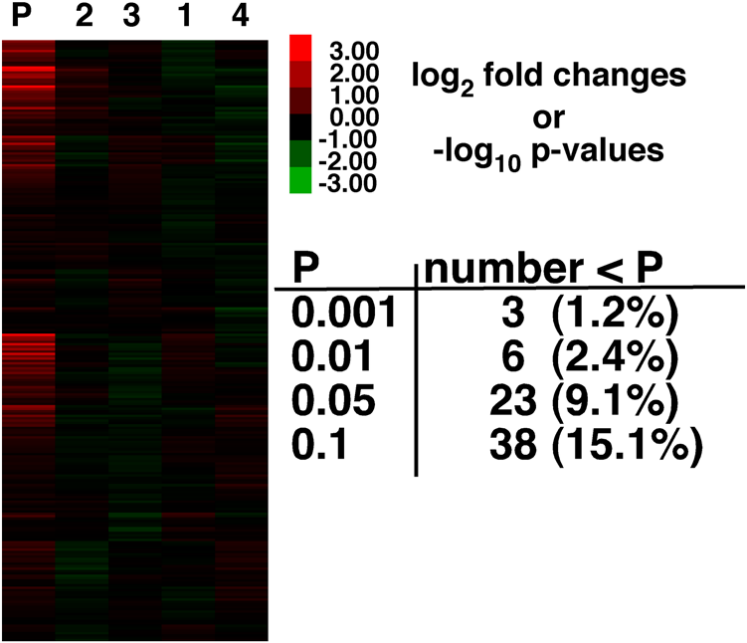
Gene expression for Single Blood isolate. Heat map for single blood isolate that was divided into four. RNA was purified, labeled and hybridized separately for each sample. Red is greater and green is less than the average gene specific fluorescence. First column (P) is the p-value from a one-way ANOVA. Only 6 genes (2.3%) out of 252 are significant at a critical p-value of 0.01. P-values (−log_10_) shown in the heat map are from an ANOVA for significant differences among samples using the 8 replicates for each separate RNA isolation. Color bar gives fold difference for log_2_ gene expression (e.g., 2 = 4×) and negative log_10_ p-value (e.g., 2 = p-value of 0.01).

A one-way ANOVA was used to test for the technical variation in gene expression between the four RNA samples isolated from a single blood sample (Fig. 2). Among all 252 genes (eight replicates per gene per sample) only 6 genes were significantly different for the four isolates at a critical p-value of 0.01. Three false-positives are expected at a p-value of 0.01 and thus with only 6 significant differences (Fig. 2) there is little evidence that separate RNA isolation, labeling and hybridization has much affect on measures of gene expression. The lack of differences is not due to high technical variation: CV (standard deviation/mean) among the eight replicates was 4% and, only three genes had a CV of >10%. Nor was it due to the low p-value of 0.01 *versus* 0.05 (Fig. 2); the number of significant differences simply reflects the p-values.

**Figure 3:**
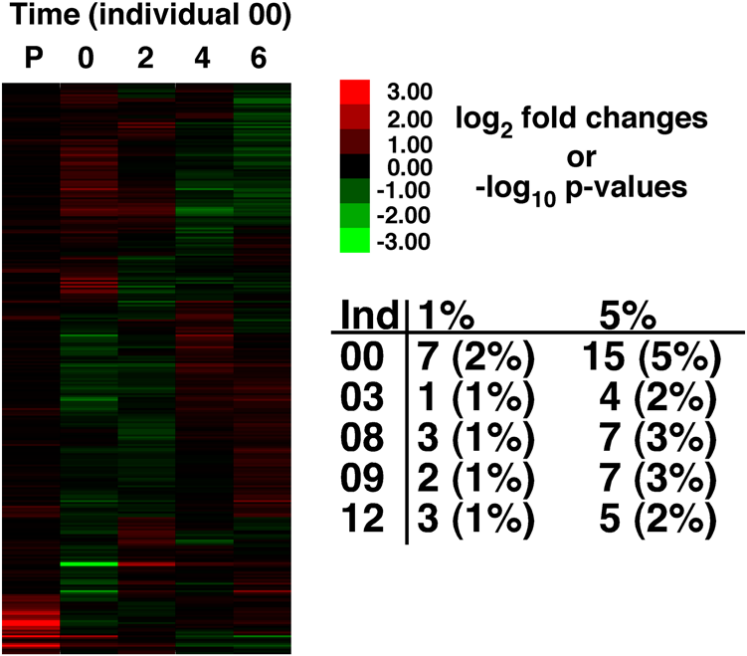
Individuals sampled over time. Heat map for one individual (00) sampled 4 times over a total of 6 weeks. Numbers above the heat map are time points (0, 2, 4 & 6 weeks) and the “P” is for p-value (−log_10_). P-values are from the ANOVA that tested for differences among separate blood isolations within an individual (4 isolations and 8 replicates per isolation). For gene expression, red is greater and green is lower expression than the mean expression for each gene. Table provides number of significant genes and percent (rounded up) out of the total of 304. Color bar gives fold difference for log_2_ gene expression (e.g., 2 = 4×) and negative log_10_ p-value (e.g., 2 = p-value of 0.01).

Random biological variation can contribute to differences in expression. We tested for random biological variation by bleeding the same five individuals four times with two weeks between bleedings (Fig. 3, MIAME, TBA). For each of the 304 genes that met our criteria, an ANOVA tested for differences in expression among the four different time periods for each individual (four sample periods with eight replicates per gene per sample period). Among the four temporal samples, there were between one and seven genes that had a significant difference in expression at a p-value of 0.01 (Fig. 3). Only one individual had more than the expected number of false positives at the critical p-value: individual-00 had 7 (2%) significant genes at p-value 0.01 for 304 genes.

## Discussion

Understanding sources of variation in gene expression is important for determining the biological importance of measured differences in mRNA expression. The analyses of technical variation in the metabolic *F. heteroclitus* cDNA microarray suggest that measures of gene expression using the *F. heteroclitus* 384 cDNA microarray are quantitative and precise. This conclusion is based on the observation that there is a linear increase in fluorescence with increasing mRNA (Fig. 1), and that there is little additional variation due to RNA processing (Fig. 2) or the day on which RNA is isolated (Fig. 3).

There is a linear increase in fluorescence with increasing mRNA for 98.5% of genes between 0.1× to 10× concentrations (0.09 pmol/ul to 9.3 pmol/ul) and 95% of genes between 0.1× to 50× (0.09 pmol/ul to 47 pmol/ul). The linear relationship between RNA and fluorescence is quite strong for RNA concentrations of 0.1× to 10× having average R^2^ values of 0.97, and most genes (88%) have R^2^ values greater than 0.95 for these four concentrations. The genes most affected by biological saturation do not have a high fluorescence; if anything, they are less than the average (genes with R^2^<0.8 for 1× to 50× have a mean that is 60% of the mean for all other genes). The two possible explanations for biological saturation with low fluorescence are that the synthesis of amino allyl labeled RNA for these genes is strongly truncated or that there is less DNA printed on the array for these genes. Truncation of amino allyl labeling would produce many more short probes with few labels per probe. Thus, to produce a similar fluorescence many more molecules would be necessary and these would saturate the DNA on the array. These problems can be avoided by using moderate amounts of probe (<10 pmol/ul). We typically avoid this problem by using 0.7 to 2 pmol/ul. Using concentrations of RNA up to 50× (47 pmol/ul) is feasible, but our data suggest that at this high of a concentration some genes will biologically saturate the cDNA on the array and therefore should be avoided.

If RNA samples are amino allyl labeled using one round of T7-RNA synthesis [22] versus two rounds of T7-RNA synthesis, 11% of genes have significant differences in fluorescence at a p-value of 0.01. Although this difference in gene expression for single *versus* double labeling is not large, it may be unacceptably high. Thus, we would suggest that for any one experiment that a researcher uses only single or double labeling procedures but not both within an individual experiment. Approximately half (59%) of genes with a significant difference between single and double labeling were greater for single labeling. The greater fluorescence for single labeling than that for double labeling would occur if cDNA or RNA synthesis was truncated with each round of labeling. Truncation would occur if the synthesis of cDNA or RNA were incomplete forming shorter nucleotide sequences with less fluorescence per RNA.

We used blood to test the effect of different RNA isolations, amino allyl labeling and hybridizations. The first experiment used a single blood isolation that was divided into four equal samples. There are few differences in expression, 2.4% at a p-value of 0.01 (i.e., six *versus* the expected three false positives). If a Bonferroni correction was applied none of these genes would be significant. Therefore, technical errors do not necessarily contribute significant amounts of variation. Similar conclusions were made about microarray results among laboratories: many different laboratories yielded similar results using different varieties of platforms [13]–[15], [33]–[36]. However, a few laboratories yielded different results. Together these data suggest that good experimental practice can minimize the effect of technical variation.

In a separate experiment, five individuals were bled once every two weeks during a six-week period, resulting in four serial blood samples from each individual. Any differences in expression among sampling times could be due to technical variation, of which there is very little as shown by the previous experiment, or biological variation. That is, although fish appeared healthy, had normal blood glucose, and the stress hormone, cortisol, did not vary significantly (p>0.1), gene expression could vary significantly for unknown biological reasons. Yet, for the five individuals there are few, if any, meaningful differences in gene expression (only one individual had more than the expected number of false positives, Fig. 3). These data confirm the observation that technical errors do not necessarily affect microarray measures. Importantly, these data also suggest that for a tissue or blood sample there is little random stochastic variation in gene expression. These data are in contrast to other publications suggesting that mRNA expression is noisy and has large stochastic variation [37], [38]. The important distinction is that for a single cell, transcription is pulsatile, occurring in bursts [37], [38], and for an individual cell this creates large stochastic variation in mRNA expression. However, our results demonstrate that for millions of cells, this variation is not apparent across a 6-week time course. We suggest that if there is a large stochastic variation in each cell, sampling of millions of cells masks this variation such that the amount of expression from any one gene is stable over time.

The microarrays used here have array elements for essential metabolic genes (Table 1) and are similar to the array elements used in previous work demonstrating larger inter-individual variation in gene expression [1], [9], [16], [17]. While the data presented here addresses the sources of variation in many microarray experiments, the lack of temporal variation in gene expression in our study may only reflect the expression of the metabolic genes. However, these results are similar to studies of gene expression in humans where the same individuals were sampled over a time period of 24 hours to four weeks [39]–[41]. These studies also found relative stable expression of a more diverse set of genes when the same individuals were sampled over time. Thus, although there are good biochemical or molecular reasons to expect stochastic variation in gene expression, this variation is not necessarily observed using routine sampling methods.

Microarrays are a useful technology for observing differences in gene expression and data extracted from microarrays can be reliably reproduced. With reasonable care, any experiment involving microarrays is capable of obtaining biological data that is not masked by technical variation thereby providing a true representation of the transcriptome under a particular set of conditions. However, caution is required before making conclusions about the biological nature of the data until the sources of technical variation are understood.
